# Paper-based CRP Monitoring Devices

**DOI:** 10.1038/srep38171

**Published:** 2016-12-02

**Authors:** Shang-Chi Lin, Chung-Yuh Tseng, Po-Liang Lai, Min-Yen Hsu, Shueh-Yao Chu, Fan-Gang Tseng, Chao-Min Cheng

**Affiliations:** 1 Institute of Nanoengineering and Microsystems, National Tsing Hua University, No. 101, Section 2, Kuang-Fu Road, Hsinchu 300, Taiwan; 2 Department of Orthopaedics, Taichung Veterans General Hospital, No.1650, Sec. 4, Taiwan Blvd., Xitun Dist., Taichung City 407, Taiwan; 3 Department of Orthopedic Surgery, Bone and Joint Research Center, Chang Gung Memorial Hospital at Linkou, College of Medicine, Chang Gung University, Taoyuan, Taiwan; 4 Department of Ophthalmology, Taichung Veterans General Hospital, No. 1650, Sec. 4, Taiwan Blvd., Xitun Dist., Taichung City 407, Taiwan; 5 Department of Engineering and System Science, National Tsing Hua University, Hsinchu 300, Taiwan; 6 Institute of Biomedical Engineering, National Tsing Hua University, No. 101, Section 2, Kuang-Fu Road, Hsinchu 300, Taiwan

## Abstract

Here, we discuss the development of a paper-based diagnostic device that is inexpensive, portable, easy-to-use, robust, and capable of running simultaneous tests to monitor a relevant inflammatory protein for clinical diagnoses i.e. C-reactive protein (CRP). In this study, we first attempted to make a paper-based diagnostic device via the wax printing method, a process that was used in previous studies. This device has two distinct advantages: 1) reduced manufacturing and assay costs and operation duration via using wax printing method to define hydrophobic boundaries (for fluidic devices or general POC devices); and, 2) the hydrophilicity of filter paper, which is used to purify and chromatographically correct interference caused by whole blood components with a tiny amount of blood sample (only 5 μL). Diagnosis was based on serum stain length retained inside the paper channels of our device. This is a balanced function between surface tension and chromatographic force following immune reactions (CRP assays) with a paper-embedded biomarker.

Advances in real-time monitoring have enhanced clinicians’ ability to provide timely diagnoses via such techniques including pregnancy tests, strip tests (i.e. nitrite, bovine serum albumin, pH), and glucose metering^1^. Unfortunately, not all clinical assays can employ rapid detection, and conventional “point-of-care” (POC) diagnostic testing to determine disease occurrence or disease state in a timely manner at or near the site of patient care requires significant manpower, materials, effort, instruments, time, and expenditure. When a POC assay must be executed under strict conditions, early detection and treatment are unlikely, especially in developing or underdeveloped regions. This exacerbates the quality of care, including follow-up care, and opens the door to medical resource and therapeutic drug misuse and waste.

Such Technology is often lacking or difficult to implement in developing countries, war zones, and home care settings^2^,^3^,^4^,^5^,^6^. Thus, there is an urgent need for POC diagnostics that are inexpensive and easy to implement in a wide variety of settings. According to the World Health Organization (WHO), *in vitro* diagnostic devices intended for use in developing nations (resource-limited areas) should be reliable, affordable, sensitive, specific, user-friendly, rapid, robust, equipment-free, and deliverable to end-users^7^. Correct diagnosis is critical for optimal therapy. Clinicians must often observe real-time clinical information to adequately diagnose a disease, thus real-time clinical care is an important aspect of rapid and easy-to-use POC diagnostic systems.

Low-cost, paper-based diagnostic systems built to detect (monitor and quantify, in advance) clinical data have been widely demonstrated in various research communities including analytical chemistry, clinical chemistry, and ELISA assays on a chip (lab on a paper); such devices can be used in developing countries or regions that suffer from energy shortages^8^,^9^,^10^,^11^,^12^,^13^,^14^. The clinical samples successfully tested using paper-based devices include urine, saliva, and whole blood^15^,^16^,^17^. The processing of whole blood samples can be challenging because whole blood requires purification processing (e.g., filtration, centrifugation) to avoid unwanted background noise during analysis. However paper-based materials can be used for purification and chromatographic processes that improve desired signal mechanisms^18^. Analytical results from these devices can be compared to results from a commercially available C-reactive protein (CRP) measuring machine (hitachi-7600) for paper-based device calibration.

Our novel paper-based device employs an inflammation detection method using a latex agglutination reaction with CRP, resulting in a quantifiable reaction (stain length) that can be observed with the naked eye. CRP is secreted by the liver, and rapidly rises to acute levels that are 10–100 times the normal range (acute-phase inflammation) in patients with trauma, ischemia, burns, inflammation, or infection^19^,^20^,^21^. Expression of CRP can be used to identify and track infection and diseases that cause inflammation such as, but not limited to, rheumatoid arthritis, cardiovascular disease, and inflammatory state^22^,^23^,^24^. In our experiment, we obtained clinical samples from osteomyelitis patients and used the above-described detection methods to monitor inflammation in patients of varying health status (ranging from critical to stable) to facilitate timely application of optimal treatment protocol.

Our paper-based diagnostic device CRP assays were used to measure serum stain length retained inside a paper microchannel. Serum stain length was affected and determined by an aggregative reaction, i.e., C-protein reacting with commercial ASK^®^ LATEX, and the increased viscosity of blood containing high levels of protein. Test results provide a preliminary assessment and can be semi-quantified for rapid assessment. Because blood agglutination is induced by the immune reaction between related antigens in the blood sample and the antibody immobilized in the paper substrate of the device/channel, the strength of agglutination can be quickly determined (5 minutes) by measuring the length of the resulting blood sample stain in the microchannel of our device while surface tension and siphon force are in balance. A shorter length indicates a stronger immune interaction and greater agglutination. Because the elapsed detection time for CRP assays using our paper-based diagnostic device (about 5 minutes actual assay time per assay) is less than the elapsed detection time for traditional methods (about 3–4 hours actual assay time per assay), the waiting time for laboratory data is shortened and the efficiency of clinical practice may be improved (Fig. 1). Note, traditional processing methods require additional time due to a number of human processing factors and can vary from country to country, but is approximately 1 day in clinical practice.

## Results

### Fabrication for Paper-based diagnosis devices

Paper, cotton, and cloth, have been successfully used as substrates for biochemically based reactions such as enzymatic reactions or antibody-antigen recognitions. For our paper-based device, we used a commercial wax printer, as opposed to SU-8 photolithography, to create fluidic channels^9^,^25^,^26^. In this manner, we built a low-cost *in vitro* diagnostic device using filter paper as the substrate as shown in Fig. 2a,b.

Before clinical sample analysis, we examined and characterized the properties of the patterned paper device being used. Primarily, we needed to confirm hydrophobicity of the wax used following the heating process and consider adjusting heating conditions to ensure the creation of adequate and consistent hydrophobic boundaries. The contact angles of the wax boundaries following wax application, heating, and drying, were approximately 100°–120°, and the wax boundaries were created when the wax/substrate composite was heated at 95 °C for 10 minutes (Figure S1). It is important to emphasize that wax must fully penetrate, i.e., melt through, the paper in order to properly define hydrophobic and hydrophilic boundaries on both sides of the filter paper (note filter paper does not retain undissolved resin in the hydrophilic regions), as shown in Fig. 2a,b and Figures S1 and S2. To confirm channel uniformity, we dropped deionized water (DI water) into three channels of each device and examined fluid flow time to determine device yield rate (Figure S2). At present, we have a fabrication yield rate of 12–14/20 (approximately 60–70%) devices when using Whatman filter paper grade 4.

### Paper-based diagnosis devices for CRP assays

In clinical practice, CRP level is routinely used as a biomarker to determine inflammation/disease state. In normal individuals, CRP level is approximately 0.02–1.35 mg/dL as measured using a commercial latex reagent. In patients experiencing acute disease response and associated inflammation, the average CRP level is 4.7 mg/dL; this level may rise to more than 40 mg/dL^27^,^28^. To extend the detection utility of our paper-based diagnostic device, we impregnated our paper with clinically used commercial latex reagents to determine CRP concentration in whole blood and create a highly useful POC tool for measuring and monitoring inflammation and tissue damage (see Fig. 2c and movie). Day-to-day variations in CRP concentration are highly useful for indicating inflammation level and disease or therapy progress. For this reason, frequent testing is highly advisable, but typically expensive and time-consuming.

When using whole blood, we observed that blood flow stops but serum continues to flow for a short time. We used total located length blood flow, including serum flow, in each channel to define serum stain length during CRP-assays analysis (naked eye measurement) (Figure S3). Figure 2d displays promising preliminary results with one positive, one negative control, and one sample channel (at the normal clinical level of CRP in whole blood). Due to serum agglutination resulting from the immune-reaction between the CRP in the serum with Ca^2+^ and the latex immobilized in the paper channels, the strength of agglutination can be determined in 5 minutes based on the blood sample stain length within the channel, which is a balance between surface tension and chromatography; shorter length equates to stronger immune interaction with greater agglutination and high concentration of CRP. We confirmed the length deviation (LD) and length ratio (LR) qualitative capacity of our paper-based diagnostic device for CRP analysis by comparing flow length deviation (naked eye) between the negative channel (NC) and the sample channel (SC) or between the sample channel (SC) and the positive channel (PC) (Fig. 2d). The correlation between LD and CRP concentration using our paper-based device is shown in Fig. 3 and the correlation between LR and CRP concentration using our paper-based device is shown in Fig. 4. Our results indicate that LD had good values for R-Square: 0.775 and Pearson’s r: 0.883 for creating a good standard curve and LR had good values for the residual sum of squares: 0.330 and Pearson’s r: −0.588, demonstrating a moderately correlated parameter.

Erythrocyte sedimentation rate (ESR) measurements are often replaced by CRP measurements in clinical monitoring. However, studies have found that ESR and CRP measurements have an inseparable relationship and the examination of both is useful in the diagnosis of acute maxillary sinusitis according to independent testing^29^. In Fig. 5, we show the relationship between CRP and ESR levels though LD and LR qualitative capacity in osteomyelitis patients. When examining ESR, we observed that the qualitative capacity of LR was greater than qualitative capacity of LD (Fig. 5b, LR residual sum of squares: 0.351 is better than LD residual sum of squares: 32109.263).

Visual observation of length ratio for qualitative CRP examination in whole blood samples when using our paper-based device achieved was comparable to commercial CRP assay results, but we contend that our device provided clearer results with regard to length quantization, and our device is easier to use and more rapid than a commercial CRP assay because it does not require any additional purification process. These results support the idea that our paper-based device is more suitable for detection of whole blood samples (Fig. 6a and b), but the approach could be further improved by optimizing fabrication, sample preparation, reagent uniformity, and analytical methodology.

## Discussion

We suspected that yield rate could be improved with more uniform wax application and found that an oven, rather than a hot plate, produced more uniform wax application (channel uniformity) and a better yield rate: 12/20 (approximately 60%) to 14/20 (approximately 70%). These efforts were applied to further reduce cost and improve yield.

**Figure 1:**
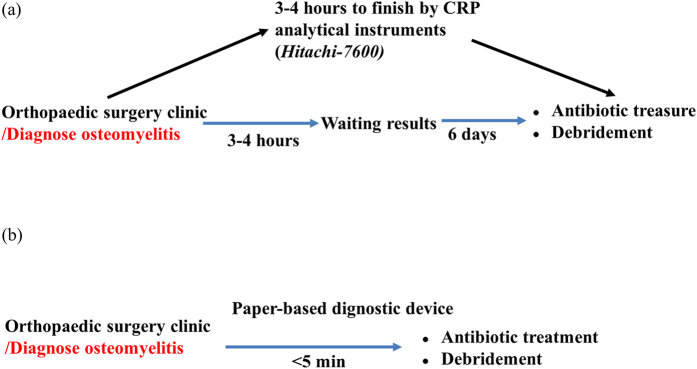
A comparison between standard clinical analysis and paper-based CRP assay device analysis in osteomyelitis patients. (**a**) The traditional process (Hitachi 7600) required approximately 3–4 hours to obtain results. (**b**) The paper-based process required approximately 5 minutes.

Regarding the uniformity of latex reagents used for paper-based devices, latex is a heterogeneous liquid that can be dropped onto the center of each channel to ensure uniform flow channel placement. During sample preparation, physiological state of the patient can affect the viscosity of blood. If blood viscosity is high, analytical results are affected. Viscous blood flows a shorter length and requires more time for flow results, which complicates analysis. For this reason, whole blood may be diluted with PBS to improve flow before being applied to a paper-based device channel. When using diluted blood samples, the volume of the sample must be carefully controlled to not exceed ~5 μL, which would overcome device design capacity. We initially designed our control group channel to contain no reagent, but when blood was applied it flowed directly to the bottom of the channel. Because this did not provide a value for comparative analysis of samples, we elected to use a three-channel/three reagent concentration system. This allowed us to test for redundancy and provided fallback options if one of the channels proved to be problematic or non-functioning. In such cases, one of the other channels could still be used to detect and identify whether a single set of tests could reliably measure the same clinical blood sample and provide a successful profile comparison of flowed serum length as follows: negative channel ≧ sample channel > positive channel (Fig. 1d). Comparing LD and LR, we found that LD was more suitable for creating a standard curve using our paper-based device.

We found that whole blood samples sometimes demonstrated hemolysis (we compared natural red blood cell sedimentation and yellow serum in our collection tube), which would seriously affect the accuracy of our experimental testing due to blood cell protein interference. We further speculated that, if problematic hemolysis was present, a paper-based diagnostic device would only be useful if it could detect its target before hemolysis occurs. If the results of this device can be validated and the underlying mechanism can be quantitatively proven, its test results may be admissible in clinical practice. For this reason, we suggest same-day use of whole blood in experiments using paper-based diagnostic devices. Interference may also be avoided by using heparin as an anticoagulant agent, rather than EDTA, because EDTA binds divalent ions. Other scientists may find novel means of avoiding interference signaling that may include permitting blood cell coagulation at the microchannel initiation point so that only serum subsequently flows through the channel for analysis^15^.

CRP assays using whole blood can be complicated. The amount of protein being detected is relatively small, but whole blood is complex and composed of many substances. In our experiments, natural filtration and chromatographic processes via the paper reduced the background “noise” of these substances and provided us with a 62.5% (45/72) success rate for qualitative CRP detection. Although our results could not be perfectly quantified, we believe the qualitative analysis provided to be useful. It is important to note that CRP background levels must be known when using CRP to interpret inflammation or tissue damage. CRP level alone has little value as a diagnostic tool especially in an emergency setting. Additional tests or assays must be conducted to confirm the significance of CRP data: a rise in CRP indicates that there is a problem but it cannot be used to establish a final diagnosis. For example, the diagnosis of a patient with conditions such as osteomyelitis involves the measurement of CRP and ESR^30^,^31^. High levels of CRP and ESR indicate an underlying clinical status. High CRP levels indicate acute inflammation and elevated ESR levels are indicative of chronic inflammation^31^. CRP can be detected in assays that take advantage of protein binding with latex in the presence of calcium ions, resulting in rapid agglutination, and ESR is measured by noting blood cell sediment height per hour (mm/hour). As inflammation initiates or blood infection rises, CRP rises rapidly, but other biomarkers, e.g., ESR, must be assayed to support the detection of inflammation. To assay for ESR, anticoagulant sedimentation tubes containing blood sample are placed upright for one hour, and travel rate and plasma concentration are observed. ESR is affected by many factors, the most important of which is the formation of cord-like erythrocytes. Because red blood cells aggregate, stack, and create rouleaux formations, exposure to plasma resistance is reduced, and the rate of decline is much faster than the rate of decline for single red blood cell dispersion^32^. Assay methods that employ filtration and chromatography, as opposed to sedimentation, allow whole blood to be separated into blood cells and serum and, as with our paper-based process, travel characteristics can be observed. Because our approach is similar to conventional ESR assay techniques, we believe that our device may be correlated to conventional ESR (Fig. 5b).

We developed a novel paper-based diagnostic device that employs hydrophilic and hydrophobic layers of wax to define a lateral flow channel. This device may be used with integrated chromatography and filtration to determine CRP level (qualitative and preliminary, semi-quantitative). As an alternative to existing conventional techniques, our paper-based diagnostic device possesses several advantages: (1) only small liquid-type sample volumes are required (~5 μL) compared to volumes required by conventional methods (~1–2 mL); (2) operating duration is short; and, (3) manufacturing and assay costs are low.

## Methods

### Fabrication of paper-based diagnostic devices

We made a paper-based diagnostic device using a wax printing method^1^. Specific patterns can be easily designed using commercial software (Autocad 2010) and printed out onto commercial filter paper (Whatman Grade No. 4 filter paper) using a commercial wax printer (Xerox Phaser 8570 DN). After wax-printed paper device fabrication we defined paper/wax performance parameters to include adequate contact angle and flow velocity measurements from three channels. Contact angle represents applied wax hydrophobic interface uniformity, and constancy and uniformity of flow confirm the flatness of the flow channel. The wax-printed paper was subsequently heated on a hot plate at a temperature of approximately 95 °C, which re-melted the wax (~10 minutes) and allowed it to penetrate the depth of the paper.

### Immobilization of CRP paper-based diagnostic devices

**Figure 2:**
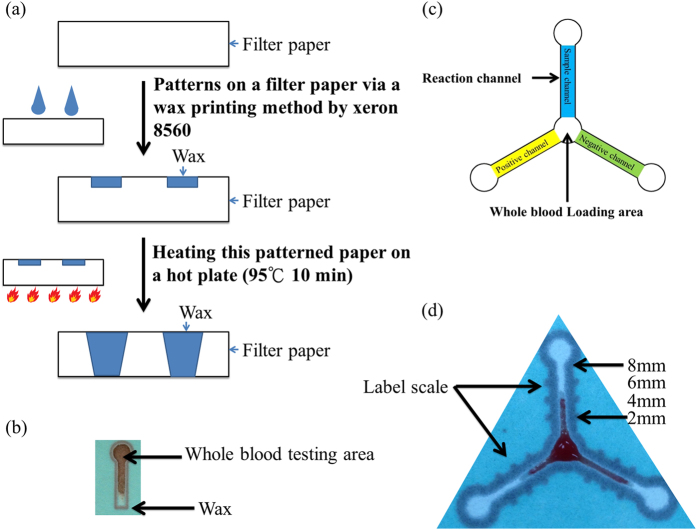
The fabrication and design of our paper-based CRP monitoring devices. (**a**) Fabrication of the paper-based diagnostic device via wax printing. (**b**) An optical image of the paper-based diagnostic device with one channel for whole blood testing. (**c**) Schematic drawing of our paper-based diagnostic device designed for CRP assays (negative channel, sample channel, positive channel – a gradient for reagent concentration). Latex was loaded from the 3 outer circles for immobilization. Blood sample was loaded into the center circle for testing. (**d**) Actual picture of experiment result shows the profiles of one CRP assay on our paper-based diagnostic device (paper chromatography).

**Figure 3:**
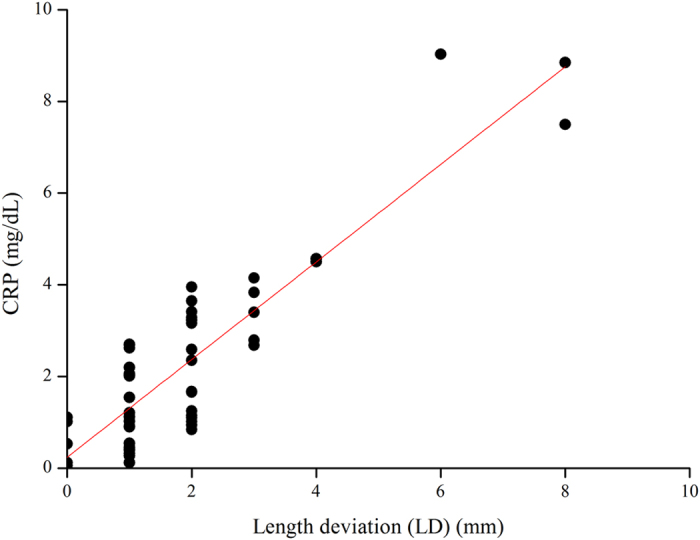
The length deviation (LD) results of paper-based device CRP analysis using diluted blood. Visual determination (naked eye) of blood flow length for serum of blood samples spotted onto our paper-based device using a measuring stick (smallest unit of measure 1 mm) and CRP concentration as measured via conventional means (Hitachi 7600). The curve fitting to the data using the equation display Adj. R-Square: 0.775 and Pearson’s r: 0.883.

**Figure 4:**
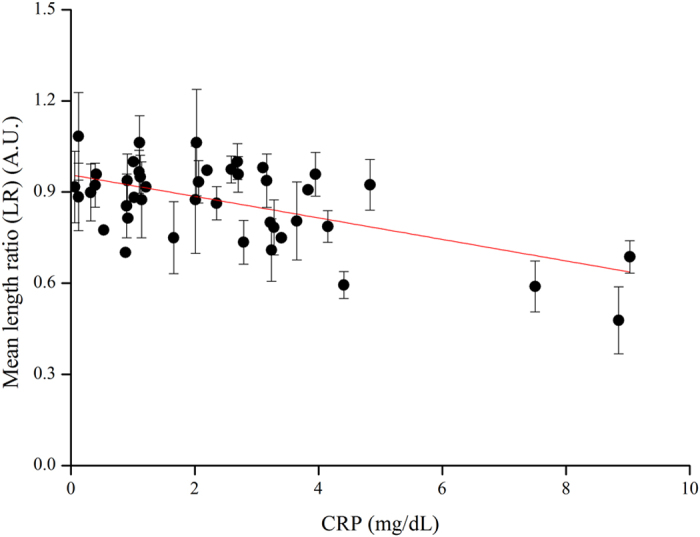
The length ratio (LR) results of paper-based device CRP analysis using diluted blood. Visual examination of spotted serum length on our paper-based device. CRP concentration as measured via conventional means (Hitachi 7600). Samples number n = 3. The curve fitting to the data using the equation displays R-Square: 0.330, and Pearson’s r: −0.588.

**Figure 5:**
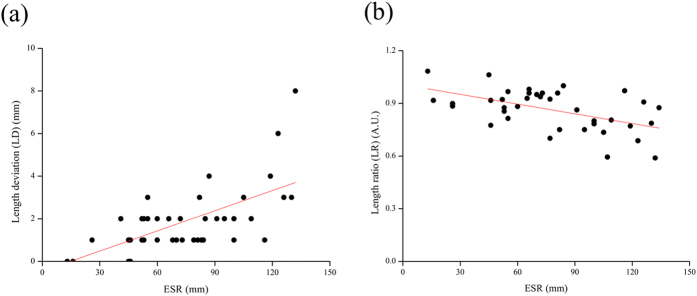
The results of paper-based device analysis of ESR showing LD and LR in diluted blood. We obtained sensitivity using a linear equation to generate an Orginpro 8.5 curve fitting. This figure shows the calibration plot for the length ratio of the results for the reagent compound (latex) reaction in our paper-based device compared to medical mainframe examinations of ESR and CRP data. (**a**) Patient samples indicating osteomyelitis inflammation by LD. (**b**) Patient samples indicating osteomyelitis inflammation by LR. The curve fitting to the data using the equation displays R-Square LD: 0.301 LR: 0.264, Pearson’s r LD: 0.563 LR: 0.533 and Residual Sum of Squares LD: 32109.263 LR: 0.351.

**Figure 6:**
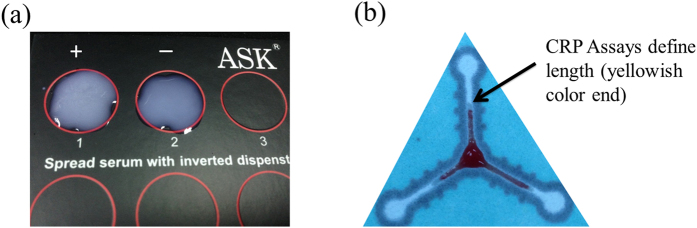
A comparison between commercial CRP assays and paper-based CRP devices. (**a**) A display of positive and negative space when observing aggregated latex, (**b**) Each CRP assay channel defines stain length from serum (yellowish color) to calculate LD or LR.

We used commercial latex (ASK^®^ LATEX agglutination test for C-reactive protein No. 06C010/6R011/06R012) for our paper-based diagnostic device CRP assays. We used a two-step procedure to perform the CRP test on our paper-based device including: 1) immobilization of ASK LATEX (negative control, sample, positive control) at different concentrations (0.75, 1.5 and 2.25 μL) by pipette at different arms of the paper tri-channels, followed by drying (~15 minutes); and, 2) placement of 5 μL diluted blood sample (blood: PBS = 1:1) onto the center of the tri-channels to initiate reaction (~10 minutes until the blood stopped flowing) (Fig. 2c,d).

### Analysis of CRP assays using paper-based diagnostic devices

For CRP assays, we examined and measured the post-reaction length of flow for applied blood samples and compared that length with negative and positive control reactions (i.e., flow lengths) to create length deviation and length ratio for qualitative CRP results (Fig. 2d).


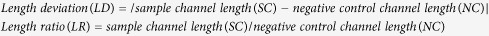


### Experimental protocol

The clinical experiment protocol was approved by the Institute of Review Board of Taichung Veterans General Hospital (Protocl number/IRB number: CF13103, 2013). This study was conducted in accordance with the ethical principles of the Helsinki declaration^33^ and International Conference on Harmonisation of technical requirements for registration of pharmaceuticals for human use-Good Clinical Practice(ICH-GCP) guideline. The inclusion criterion of this experiment was patient diagnosis of osteomeylitis and ESR and CRP were regularly checked during the admission period. Approved informed consents (CF13103, version 03) were obtained before blood samples were collected from participants. Two blood sample tubes (5cc) were collected from patients in this study. One tube was sent for laboratory study of ESR and CRP using an Hitachi 7600 CRP measuring device for comparison (control group). The other tube was used to test our paper-based diagnostic device (experimental group). A total of 72 paired samples (control and experimental sample) were collected in this study.

## Additional Information

**How to cite this article**: Lin, S.-C. *et al*. Paper-based CRP Monitoring Devices. *Sci. Rep.* **6**, 38171; doi: 10.1038/srep38171 (2016).

**Publisher's note:** Springer Nature remains neutral with regard to jurisdictional claims in published maps and institutional affiliations.

## Supplementary Material

Supplementary Movie

Supplementary Information

## References

[b1] Gubala V., Harris L. F., Ricco A. J., Tan M. X. & Williams D. E. Point of care diagnostics: status and future. Anal. Chem. 84, 487–515 (2011). 22221172 10.1021/ac2030199

[b2] Chin C. D., Linder V. & Sia S. K. Lab-on-a-chip devices for global health: past studies and future opportunities. Lab Chip 7, 41–57 (2007). 17180204 10.1039/b611455e

[b3] Sia S. K., Linder V., Parviz B. A., Siegel A. & Whitesides G. M. An integrated approach to a portable and low-cost immunoassay for resource-poor settings. Angew. Chem. 43, 498–502 (2004). 14735545 10.1002/anie.200353016

[b4] Daar A. S. . Top ten biotechnologies for improving health in developing countries. Nat. Genet. 32, 229–232 (2002). 12355081 10.1038/ng1002-229

[b5] Yager P. . Microfluidic diagnostic technologies for global public health. Nature 442, 412–418 (2006). 16871209 10.1038/nature05064

[b6] Mabey D., Peeling R. W., Ustianowski A. & Perkins M. D. Diagnostics for the developing world. Nature reviews. Microbiology 2, 231–240 (2004). 15083158 10.1038/nrmicro841

[b7] Peeling R. WHO programme on the evaluation of diagnostic tests. Bull. W. H. O. 84, 594 (2006). 16917641 PMC2627431

[b8] Martinez A. W., Phillips S. T., Whitesides G. M. & Carrilho E. Diagnostics for the Developing World: Microfluidic Paper-Based Analytical Devices. Anal. Chem. 82, 3–10 (2010). 20000334 10.1021/ac9013989

[b9] Cheng C. M. . Paper-Based ELISA. Angew. Chem., Int. Ed. 49, 4771–4774 (2010). 10.1002/anie.201001005 20512830

[b10] Ellerbee A. K. . Quantifying Colorimetric Assays in Paper-Based Microfluidic Devices by Measuring the Transmission of Light through Paper. Anal. Chem. 81, 8447–8452 (2009). 19722495 10.1021/ac901307q

[b11] Cheng C. M. . Millimeter-scale contact printing of aqueous solutions using a stamp made out of paper and tape. Lab Chip 10, 3201–3205 (2010). 20949218 10.1039/c004903d

[b12] Liu X. Y. . A Portable Microfluidic Paper-Based Device for Elisa. Proc. Ieee. Micr. Elect0. 75–78 (2011).

[b13] Martinez A. W. . Programmable diagnostic devices made from paper and tape. Lab Chip 10, 2499–2504 (2010). 20672179 10.1039/c0lc00021c

[b14] Martinez A. W. . Simple telemedicine for developing regions: camera phones and paper-based microfluidic devices for real-time, off-site diagnosis. Anal. Chem. 80, 3699–3707 (2008). 18407617 10.1021/ac800112r PMC3761971

[b15] Yang X., Forouzan O., Brown T. P. & Shevkoplyas S. S. Integrated separation of blood plasma from whole blood for microfluidic paper-based analytical devices. Lab chip 12, 274–280 (2012). 22094609 10.1039/c1lc20803a

[b16] Klasner S. A. . Paper-based microfluidic devices for analysis of clinically relevant analytes present in urine and saliva. Ana. l Bioanal. Chem. 397, 1821–1829 (2010). 10.1007/s00216-010-3718-4 20425107

[b17] Martinez A. W., Phillips S. T., Whitesides G. M. & Carrilho E. Diagnostics for the developing world: microfluidic paper-based analytical devices. Anal. Chem. 82, 3–10 (2010). 20000334 10.1021/ac9013989

[b18] Lin S.-C. . Cotton-based Diagnostic Devices. Sci. Rep. 4 (2014). 10.1038/srep06976 PMC5382709 25393975

[b19] Du Clos T. W. Function of C-reactive protein. Ann. Med. 32, 274–278 (2000). 10852144 10.3109/07853890009011772

[b20] Ugarte H., Silva E., Mercan D., De Mendonca A. & Vincent J.-L. Procalcitonin used as a marker of infection in the intensive care unit. Crit. Care Med. 27, 498–504 (1999). 10199528 10.1097/00003246-199903000-00024

[b21] Sherwood E. R. & Toliver-Kinsky T. Mechanisms of the inflammatory response. Best Pract. Res., Clin. Anaesthesiol. 18, 385–405 (2004). 15212335 10.1016/j.bpa.2003.12.002

[b22] Koenig W. . C-reactive protein, a sensitive marker of inflammation, predicts future risk of coronary heart disease in initially healthy middle-aged men results from the MONICA (Monitoring Trends and Determinants in Cardiovascular Disease) Augsburg Cohort Study, 1984 to 1992. Circulation 99, 237–242 (1999). 9892589 10.1161/01.cir.99.2.237

[b23] Ridker P. M., Hennekens C. H., Buring J. E. & Rifai N. C-reactive protein and other markers of inflammation in the prediction of cardiovascular disease in women. N. Engl. J. Med. 342, 836–843 (2000). 10733371 10.1056/NEJM200003233421202

[b24] Yeh E. T. & Willerson J. T. Coming of age of C-reactive protein using inflammation markers in cardiology. Circulation 107, 370–371 (2003). 12551854 10.1161/01.cir.0000053731.05365.5a

[b25] Lo S. J. . Molecular-level dengue fever diagnostic devices made out of paper. Lab Chip 13, 2686–2692 (2013). 23563693 10.1039/c3lc50135c

[b26] Wang H. K. . Cellulose-based diagnostic devices for diagnosing serotype-2 dengue Fever in human serum. Adv. Healthcare Mater. 3, 187–196 (2014). 10.1002/adhm.201300150 23843297

[b27] Pepys M. B. C-reactive protein fifty years on. The Lancet 317, 653–657 (1981). 10.1016/s0140-6736(81)91565-8 6110874

[b28] Werner M. Serum protein changes during the acute phase reaction. Clin. Chim. Acta 25, 299–305 (1969). 4184578 10.1016/0009-8981(69)90272-1

[b29] Hansen J. G., Schmidt H., Rosborg J. & Lund E. Predicting acute maxillary sinusitis in a general practice population. BMJ 311, 233–236 (1995). 7627042 10.1136/bmj.311.6999.233 PMC2550286

[b30] Aalto K., Österman K., Peltola H. & Räsänen J. Changes in erythrocyte sedimentation rate and C-reactive protein after total hip arthroplasty. Clin. Orthop. Relat. Res. 184, 118–120 (1984). 6705332

[b31] Husain T. M. & Kim D. H. C-reactive protein and erythrocyte sedimentation rate in orthopaedics. Univ. Pa. Orthop. J. 15, 13–16 (2002).

[b32] Pisetsky D. S. Laboratory testing in the rheumatic diseases. In: Goldman, L., Schafer, A. I., eds. Goldman’s Cecil Medicine (25th ed. Philadelphia, PA) chap 257 (Elsevier Saunders 2016).

[b33] General Assembly of the World Medical Association. “World Medical Association Declaration of Helsinki: ethical principles for medical research involving human subjects”. The Journal of the American College of Dentists 81. 3, 14 (2014). 25951678

