# Corrigendum: Paper-based CRP Monitoring Devices

**DOI:** 10.1038/srep44721

**Published:** 2017-03-16

**Authors:** Shang-Chi Lin, Chung-Yuh Tzeng, Po-Liang Lai, Min-Yen Hsu, Hsueh-Yao Chu, Fan-Gang Tseng, Chao-Min Cheng

Scientific Reports 6: Article number: 3817110.1038/srep38171; published online: 11 02 2016; updated: 03 16 2017

The original version of this Article contained errors in the spelling of the authors Chung-Yuh Tzeng and Hsueh-Yao Chu, which were incorrectly given as Chung-Yuh Tseng and Shueh-Yao Chu respectively. These errors have now been corrected in the PDF and HTML versions of the Article.

