# Supplementary material for: Paper-based CRP Monitoring Devices
Source: Sci Rep. 2016 Dec 2;6:38171. doi: 10.1038/srep38171 (PMC5133555; doi:10.1038/srep38171)
Supplement: Supplementary Information [file srep38171-s2.pdf]

Paper-based CRP Monitoring Devices

Shang-Chi Lin [#, 1], Chung-Yuh Tzeng [#, 2], Po-Liang Lai [3], Min-Yen Hsu [1, 4],

Shueh-Yao Chu [5], Fan-Gang Tseng [*, 5] and Chao-Min Cheng [*, 6]

[1] Institute of Nanoengineering and Microsystems, National Tsing Hua University, No. 101, Section 2, Kuang-Fu Road, Hsinchu 300, Taiwan

[2]Department of Orthopaedics, Taichung Veterans General Hospital, No.1650, Sec. 4, Taiwan Blvd., Xitun Dist., Taichung City 407, Taiwan

[3]Department of Orthopedic Surgery, Bone and Joint Research Center, Chang Gung Memorial Hospital at Linkou, College of Medicine, Chang Gung University.

[4]Department of Ophthalmology, Taichung Veterans General Hospital, No.1650, Sec. 4, Taiwan Blvd., Xitun Dist., Taichung City 407, Taiwan

[5] Department of Engineering and System Science, National Tsing Hua University, Hsinchu 300, Taiwan

[6] Institute of Biomedical Engineering, National Tsing Hua University, No. 101,

Section 2, Kuang-Fu Road, Hsinchu 300, Taiwan

[#] These authors contributed equally to this study

* These authors are corresponding authors.

Corresponding author; E-mail: chaomin@mx.nthu.edu.tw (C.-M. Cheng) &

fangang@ess.nthu.edu.tw (F.-G. Tseng)

Phone number: +886-(3) - 516-2402 (C.-M. Cheng)

Fax number: +886-(3)-574-5454 (C.-M. Cheng)

Address: No. 101, Sec. 2, Kuang-Fu Rd., Hsinchu 30013, Taiwan

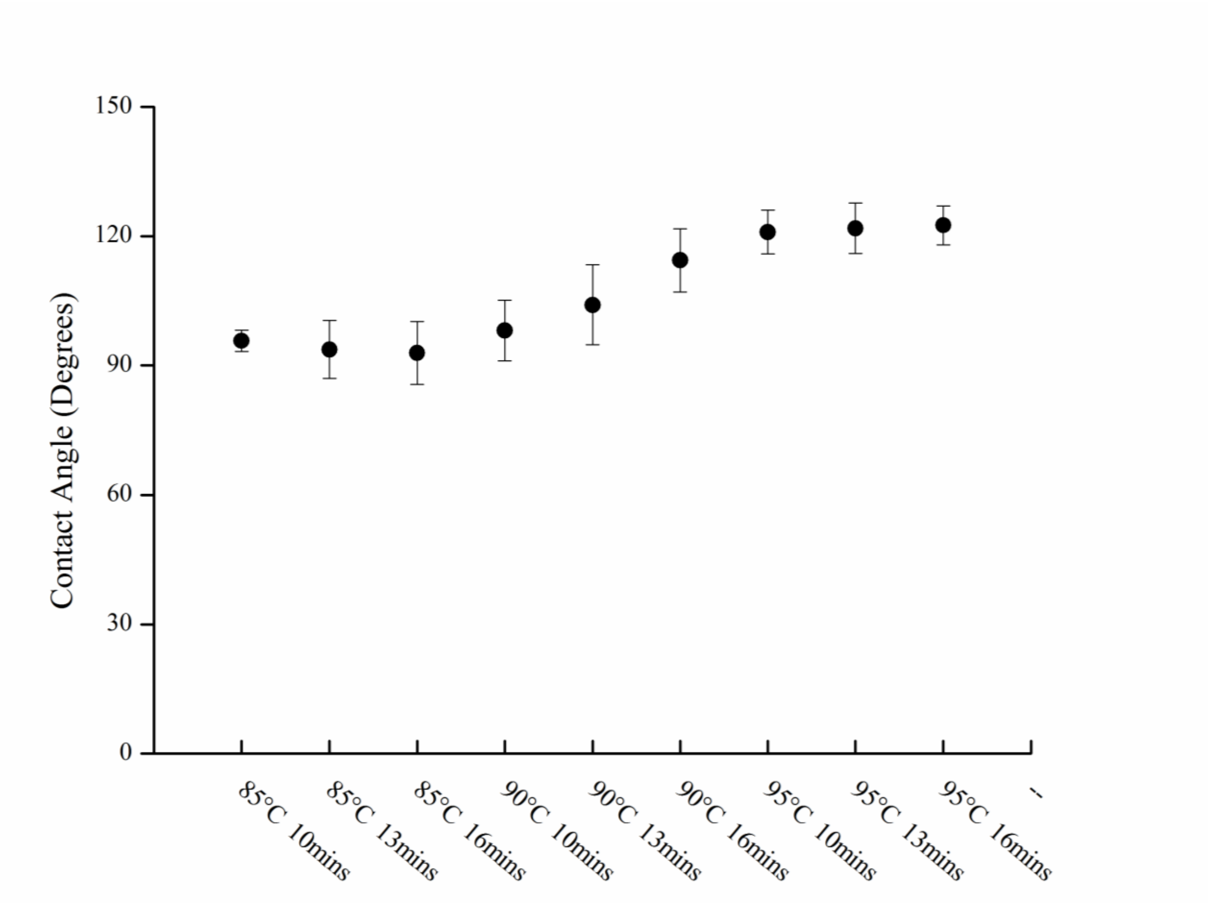

Figure S1. Contact angle results for our paper-based device using different times and temperatures. Determination of contact angle of water for our paper-based device using FTA32 analysis software. This figure shows the contact angle results for the different baking times and temperatures examined using our paper-based device as produced by Origin Pro 8.5.

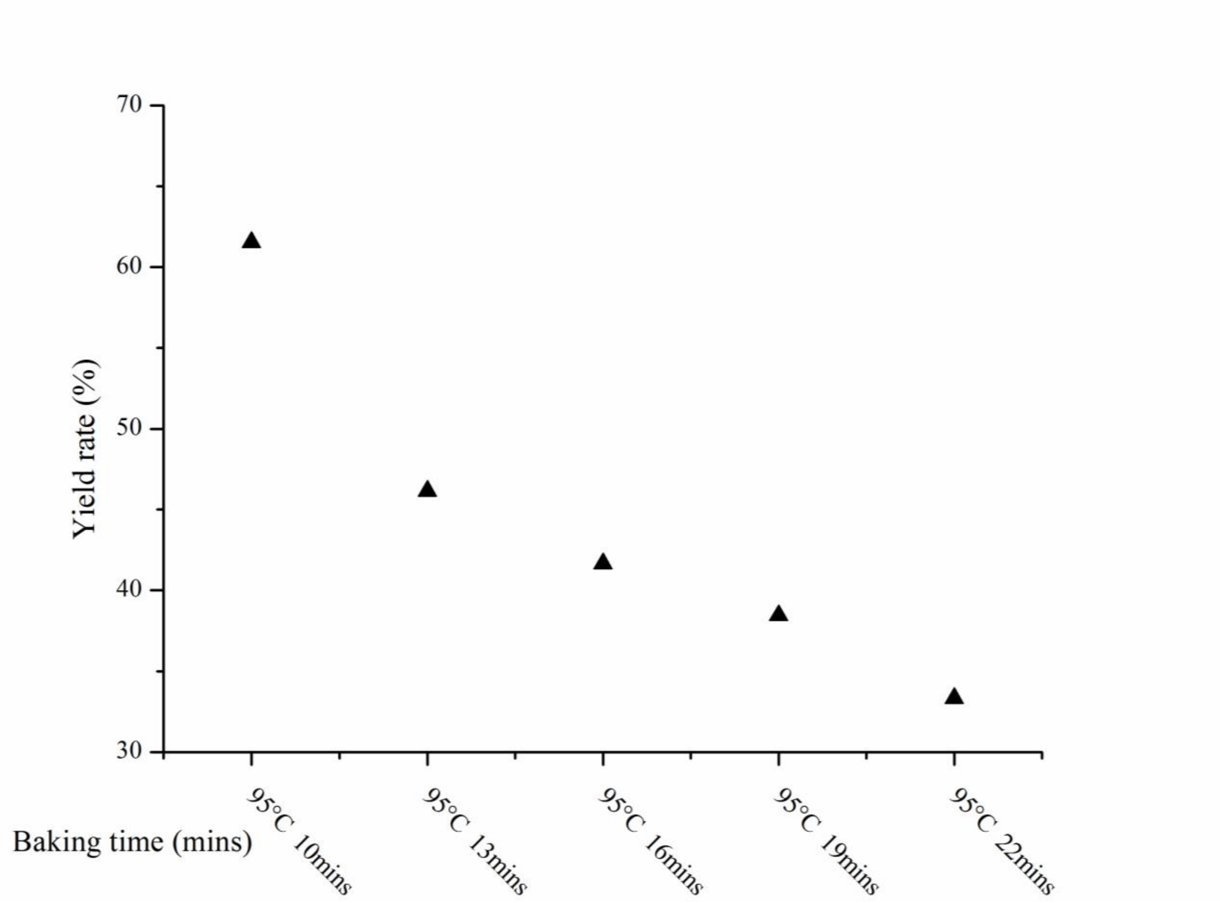

Figure S2. Yield rate results for our paper-based device using different baking times. Paper-based device yield rate was determined. This figure shows the yield rate results for the different baking times examined using our paper-based device as produced by Origin Pro 8.5.

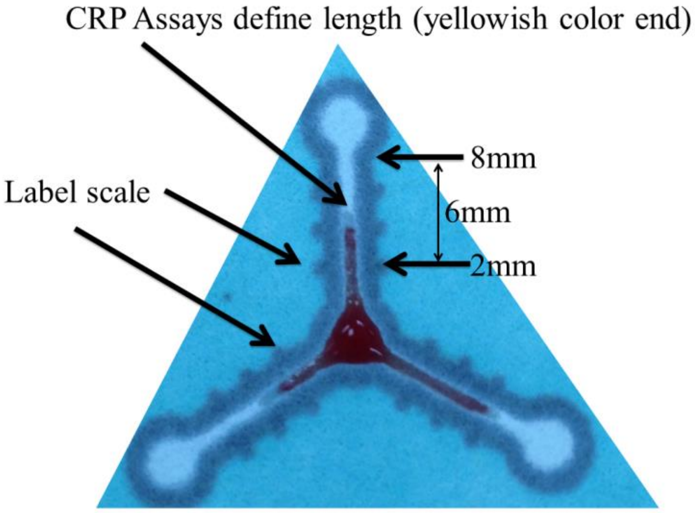

Figure S3. CRP assays using our paper-based device. Each CRP assay channel could be used to determine serum stain length (yellowish colors). Our device allowed for visual determination (naked eye) of serum stain length following the spotting of blood samples onto our paper-based device (smallest unit of measure was 1mm).

Movie. The procedure of whole blood flowing in the CRP paper based device.
